# Immunological analyses reveal an immune subtype of uveal melanoma with a poor prognosis

**DOI:** 10.18632/aging.102693

**Published:** 2020-01-18

**Authors:** Hui Pan, Linna Lu, Junqi Cui, Yuan Yang, Zhaoyang Wang, Xianqun Fan

**Affiliations:** 1 Department of Ophthalmology, Ninth People’s Hospital, Shanghai JiaoTong University School of Medicine, Shanghai, China; 2 Shanghai Key Laboratory of Orbital Diseases and Ocular Oncology, Shanghai, China; 3 Department of Pathology, Ninth People’s Hospital, Shanghai JiaoTong University School of Medicine, Shanghai, China

**Keywords:** immune subtype, uveal melanoma, bioinformatics, immune cell fractions, TCGA

## Abstract

Uveal melanoma is an aggressive intraocular malignancy that often exhibits low immunogenicity. Metastatic uveal melanoma samples frequently exhibit monosomy 3 or *BAP1* deficiency. In this study, we used bioinformatic methods to investigate the immune infiltration of uveal melanoma samples in public datasets. We first performed Gene Set Enrichment/Variation Analyses to detect immunological pathways that are altered in tumors with monosomy 3 or *BAP1* deficiency. We then conducted an unsupervised clustering analysis to identify distinct immunologic molecular subtypes of uveal melanoma. We used CIBERSORT and ESTIMATE with RNA-seq data from The Cancer Genome Atlas and the GSE22138 microarray dataset to determine the sample-level immune subpopulations and immune scores of uveal melanoma samples. The Kaplan-Meier method and log-rank test were used to assess the prognostic value of particular immune cells and genes in uveal melanoma samples. Through these approaches, we discovered uveal melanoma-specific immunologic features, which may provide new insights into the tumor microenvironment and enhance the development of immunotherapies in the future.

## INTRODUCTION

Immune heterogeneity within the tumor microenvironment has been linked to the drug sensitivity and prognosis of patients with various cancer types [1, 2]. Thus, the profiling of immune signatures might uncover biomarkers for targeted therapy and clinical outcome assessment. Recently, datasets from The Cancer Genome Atlas (TCGA) have been used to depict the immune landscapes of multiple tumor types [1]. Researchers have used integrated approaches and multidimensional datasets to determine the infiltration levels and co-infiltration networks of various immune cell populations in tumors [3, 4]. For instance, genomic data and hematoxylin & eosin image data were used to assess the total lymphocyte infiltration and immune cell fractions of the tumor microenvironment in different cancer types in TCGA [3]. This analysis revealed common immune subtypes, immune gene expression signatures and tumor-extrinsic features, which could be used to identify transcriptional regulatory networks in the tumor microenvironment. Moreover, an extensive immunogenomic analysis of PanCancer TCGA data from 33 diverse cancer types revealed six distinct immune subtypes and various tumor-immune cell interactions [1].

Uveal melanoma (UM) is the most common aggressive intraocular malignancy in adults, and originates from the uveal tract [5]. UM is characterized by different cytogenetic alterations than cutaneous melanoma (CM), and has the potential for hepatic metastasis [6]. Nevertheless, UM and CM share a common lineage that is determined by melanoma-specific neural crest genes [7]. Recently, a comprehensive analysis of 80 UM cases identified four molecularly distinct subtypes. Monosomy 3 (M3) tumors were found to be enriched for genes in immune pathways such as interferon signaling, T cell invasion and cytotoxicity [8]. Several reports have demonstrated that CM is highly infiltrated by immune cells such as CD4 and CD8 cells [9–11]. However, only a subset of UM patients exhibit similar lymphocyte infiltration of their tumors, suggesting that UM immune infiltration is heterogeneous [7]. In liver metastases, the tumor-infiltrating lymphocyte activity is lower in UM patients than in CM patients [5, 7, 12].

The inflammatory phenotype of UM is characterized by high infiltration of lymphocytes and macrophages, and by the expression of human leukocyte antigen (HLA) Class I and II antigens [13]. Mutation of GNA11/GNAQ was not found to significantly alter the immune infiltration and HLA Class I expression of primary UM [14]. However, the nuclear factor kappa-light-chain-enhancer of activated B cells (NF-kB) pathway was found to be associated with the inflammatory phenotype and high HLA Class I expression, and was upregulated upon the inactivation of *BAP1* in UM. The levels of the NF-kB pathway molecules NF-kB1, NF-kB2 and RELB were reported to correlate positively with the expression of HLA Class I and with the infiltration of T cells and macrophages in UM [15]. Moreover, one of the most significant UM studies revealed that lymphocyte infiltration and tumor-associated M2 macrophage levels were associated with a poor prognosis in primary UM after adjustment for other risk factors [16]. In another study, the authors used a digital PCR-based T cell quantification method to characterize the prognostic value of the T cell count and activated macrophage level in the microenvironment of UM [17]. Thus, characterizing the immunological features of UM may provide novel immune biomarkers for prognostic assessment and immunotherapy.

Immunotherapy through immune checkpoint blockades has displayed promising clinical efficacy in multiple tumor types [18–20]. Enhancing the cytolytic functions of infiltrating lymphocytes can significantly improve antitumor immunity [4]. However, due to the low levels of cytotoxic cells in the tumor microenvironment, non-responsiveness to immunotherapy remains a clinical challenge. Thus, a deep comprehension of the interplay among the immune cell subsets in the tumor microenvironment is needed for the development of effective antitumor immune therapies. Here, we sought to identify the molecular immune subtypes and infiltrating immune cells of UM, in order to discover possible candidates for immunotherapy.

## RESULTS

### Identification of M3/*BAP1*-specific immunological pathways using GSEA

**Figure 1:**
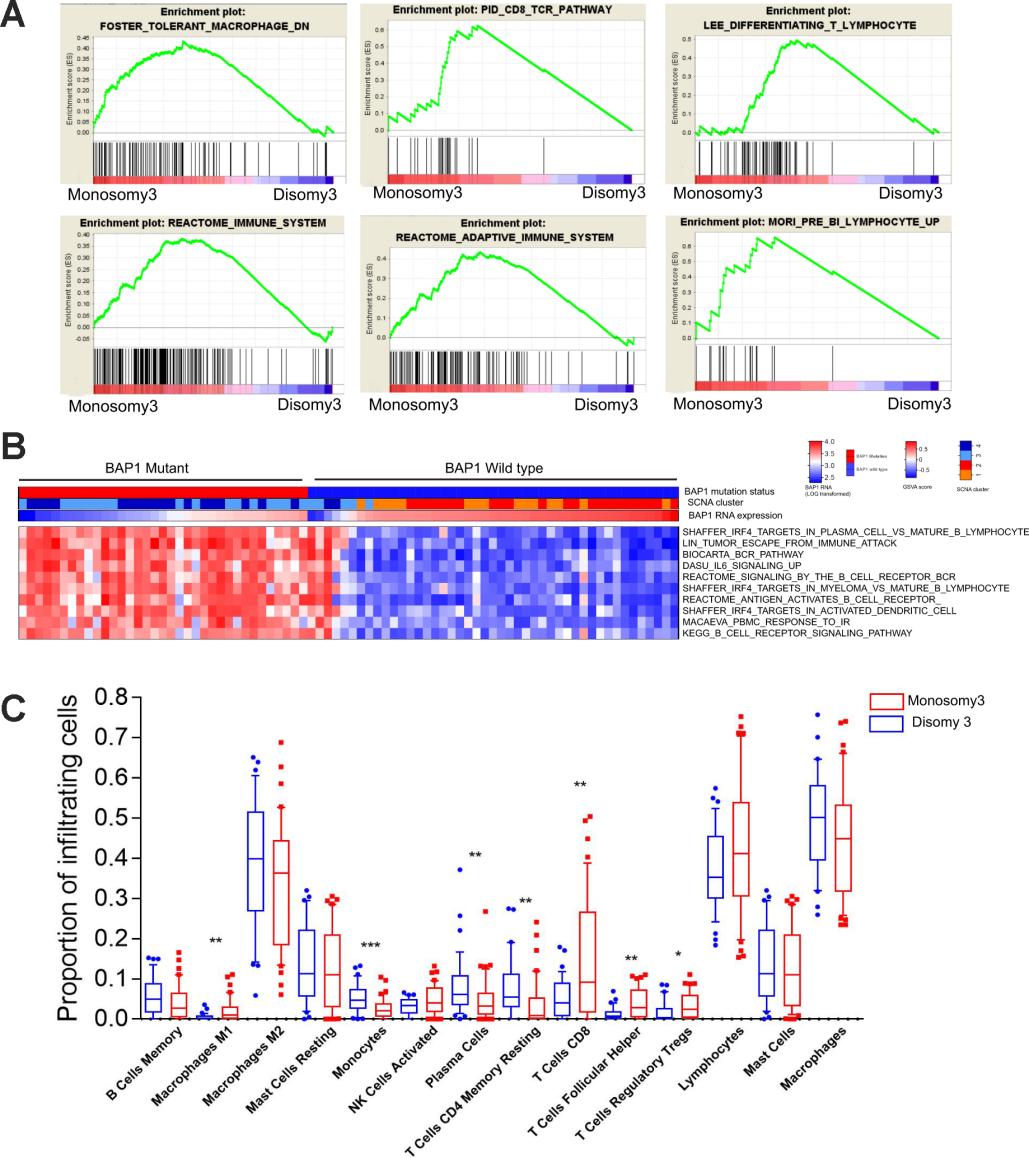
***BAP1*-mutant/M3 UM is enriched in immune signatures.** (**A**) GSEA plots of gene ontology categories, including the immune response, immune system process, adaptive immune response, immune effector process, and regulation of immune system process. (**B**) GSVA analysis of differing immune pathways between *BAP1*^wild type^ and *BAP1* ^mutant^ tumors. (**C**) Differential proportions of immune cells between D3 and M3 tumors.

*BAP1* is frequently mutated in metastatic UM, and is associated with chromosome 3 loss (M3) [16, 21]. Thus, we investigated whether *BAP1*-deficient UM samples exhibited distinct immune infiltration patterns from *BAP1*-intact samples in TCGA. Gene Set Enrichment Analysis (GSEA) was used to develop M3/*BAP1*^null^ aberrant gene signatures and reveal novel immune pathways. The immune-related gene signatures were then used to estimate the level of immune cell infiltration. Among the most significantly altered pathways in M3/*BAP1*^null^ tumors, those involving the CD8/T-cell-receptor, adaptive immune system, pre-B1 lymphocytes and differentiating T lymphocytes were significantly enriched in the M3 group (Figure 1A), consistent with a previous study [22]. In curating the differential gene expression data, we found significant gene expression changes in a variety of immune-associated processes, suggesting that M3/*BAP1*^null^ tumors highly express immune pathway genes.

Next, we performed Gene Set Variation Analysis (GSVA) to compare the sample-level infiltration of M3/*BAP1*^null^ tumors and disomy 3 (D3)/*BAP1*^intact^ tumors. The top 10 significantly enriched immune pathway gene sets were selected. As shown in the heatmap (Figure 1B), the “B-cell receptor signaling” pathway, the “IRF4 targets in plasma cells vs. mature B lymphocytes” pathway and the “peripheral blood mononuclear cell response to ionizing radiation (IR)” pathway were significantly enriched in M3/*BAP1*^null^ tumors, suggesting that IRF4 may enhance immunity in *BAP1* deficient UMs.

To further assess the immune cell subpopulations of M3/*BAP1*^null^ and D3/*BAP1*^intact^ tumors, we compared the relative abundance of immune cells between these two UM subtypes. As shown in Figure 1C, the levels of infiltrating CD8 T cells and T follicular helper cells were significantly higher in M3-subtype tumors (n = 42) than in D3 tumors (n = 38) (P<0.01, Mann-Whitney test). Of note, D3 tumors were previously reported to have a better prognosis than M3 tumors. In contrast, monocytes and CD4 memory resting cell levels were higher in D3 tumors than in M3 tumors (P<0.01, Mann-Whitney test).

### Hierarchical clustering of immune cell-associated gene expression in UM

**Figure 2:**
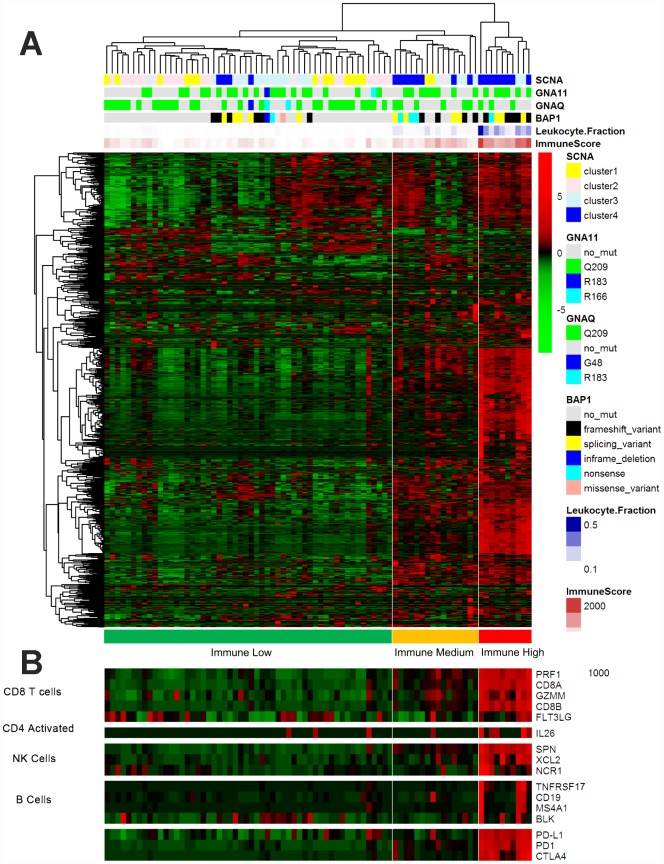
**Immune-related gene expression in the UM dataset of TCGA.** (**A**) Hierarchical clustering of 80 tumors based on 730 immune-related genes. Genes were median-centered. Each colored square represents the relative mean transcript abundance (log2 FPKM+1) for each sample, with the lowest levels shown in green, the median levels in black and the highest levels in red. The genetic mutation type, SCNA type, immune score, leukocyte fraction and *BAP1* mutation status are shown below the array tree. (**B**) The expression of selected gene signatures or genes is demonstrated below the heatmap.

We then performed an unsupervised clustering analysis of 730 immune-related genes in the UM dataset of TCGA, as described in a previous study [11]. The sample clustering revealed three clear groups of samples that separated predominantly according to the gene expression of infiltrating immune cells, here termed the Immune Low (Immune L, n=54, 67.5%), Immune Medium (Immune M, n=16, 20%) and Immune High (Immune H, n=10, 12.5%) groups (Figure 2A). As shown in the heatmap, the Immune H group expressed high levels of the majority of the immune-related genes, in contrast to the Immune L group. The Immune H group highly expressed genes associated with CD8 T cells, B cells and natural killer cells (Figure 2B). The inhibitory checkpoint molecules PD-1 and CTLA-4 and the genes directly associated with MHC CLASS I/II, Cytolytic Activity and co stimulatory Molecules were also highly expressed in the Immune H group and modestly expressed in the Immune M group (Supplementary Figure 2).

Next, we sought to determine the clinical and molecular features underlying the immune clustering. The age, American Joint Committee on Cancer stage (AJCC), sex and GNAQ/11 mutation status of the patients did not differ significantly between the Immune H group and the Immune L and M groups (Immune H vs. M vs. L). Interestingly, the *BAP1* mutation status differed significantly among the groups (P<0.0001, Fisher’s exact test, Immune H vs. M vs. L). Similarly, a significant difference in somatic copy number alteration (SCNA1/2 vs. ¾) was found among the groups (P<0.0001, Fisher’s exact test, Immune H vs. M vs. L) (Table 1). Our analysis suggested that *BAP1* mutations and chromosome alterations may determine the tumor immune state and immune infiltration of UM.

**Table 1:** Clinical–pathologic characteristics of the TCGA Dataset in this study.

**N**	**N(%)**
	**80**
Age, median (range)	60 (22–86)
Sex	
Female	35(43.8%)
Male	45(56.2%)
AJCC Clinical Stage	
II	36(45%)
III/IV	44(55%)
Histological type	
Epithelioid cell dominant	23(28.8%)
Spindle Cell dominant	57(71.2%)
GNAQ/11 status	
Mutation	74(92.5%)
Wild type	6(7.5%)
BAP1 status	
Mutation	35(43.8%)
Wild type	45(56.2%)
Monosomy3	
Monosomy3/LOH	42(52.5%)
Disomy3	38(47.5%)
SCNA	
½	38(47.5%)
¾	42(52.5%)

### Immune subtypes correlated with immune infiltration and clinical outcomes

**Figure 3:**
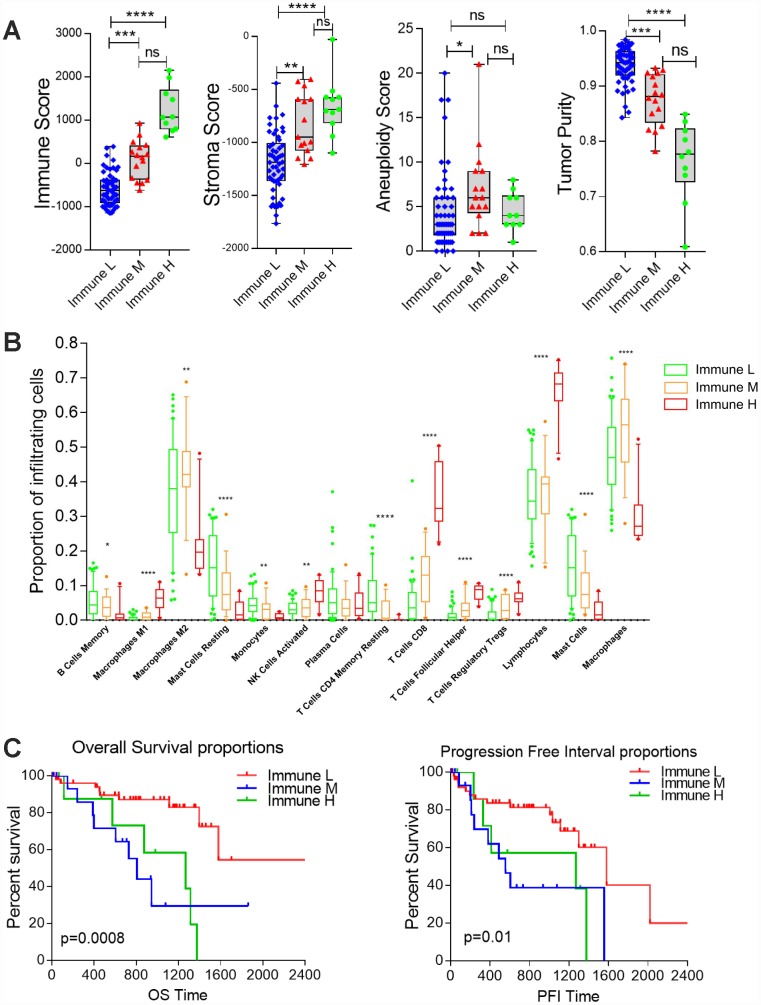
**Differential infiltration of the immune subgroups.** (**A**) Box plots comparing the distribution of immune infiltration measures in the three immune subtypes. Each box spans the interquartile range, with the lines representing the median for each group. Whiskers represent the absolute range. All outliers are included in the plot. (**B**) Differential proportions of immune cells in the immune subtypes. (**C**) Kaplan-Meier survival curves demonstrate that the Immune M and H groups of UM patients consistently exhibited worse OS and PFIs than the Immune L group (log-rank test, P<0.05).

ESTIMATE (Estimation of STromal and Immune cells in MAlignant Tumor tissues using Expression data) is a tool for predicting tumor purity and assessing stromal/immune cell infiltration into tumor tissues based on gene expression data [23]. We used the ESTIMATE algorithm to calculate the immune score and stromal score for each UM sample from TCGA, and we compared the scores of the different immune subgroups. Since the immune score reflects the overall immune infiltration based on lymphocyte gene expression, we used this score to estimate the total immune cell infiltration of each sample. The Immune L group demonstrated the lowest immune and stromal scores, whereas the Immune M and Immune H groups displayed modest and high stromal and immune scores, respectively (P<0.0001, analysis of variance [ANOVA], Immune H vs. M vs. L) (Figure 3A). The tumor purity calculated by the ESTIMATE algorithm revealed that the Immune L group was the purest subtype (mean 0.9373) and the Immune H group was the least pure subtype (mean 0.7649) (P<0.0001, ANOVA) (Figure 3A).

To further examine the tumor microenvironment, we assessed the infiltration of distinct immune subpopulations in the three tumor subtypes. The Immune L group had the lowest levels of CD8 T cells and total lymphocytes, whereas the Immune M and Immune H subtypes displayed relatively high levels of these cell types (P<0.0001, Immune H vs. M vs. L). In addition, the Immune H group had lower levels of M2 macrophages and mast cells than the other two groups. The Immune L group also exhibited low infiltration of T follicular helper cells and regulatory T cells (P<0.0001, Immune H vs. M vs. L) (Figure 3B), suggesting that there are distinct subtypes of UM with low or high T cell infiltration.

Next, we investigated the prognostic impact of immune clustering on patient survival. Interesting, we observed significantly worse overall survival (OS) and progression-free intervals (PFIs) in the Immune M and Immune H groups than in the Immune L group in the cohort from TCGA, suggesting that distinct immunological features correlate with the patient prognosis in UM (Figure 3C). However, the OS and PFI did not differ significantly between the Immune M and H groups (log-rank test, P>0.05, Immune H vs. M).

### Correlations of immune cells and immune scores in TCGA and Laurent datasets

**Figure 4:**
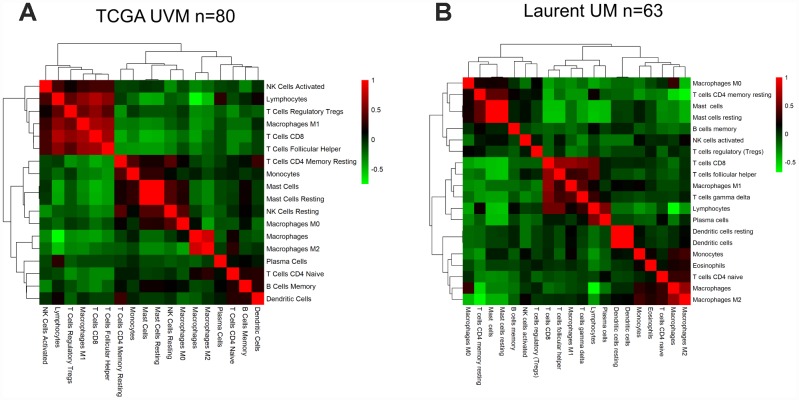
**The correlation between the immune score and immune cell infiltration in UM.** Pairwise correlation heatmap among immune cell-type scores in the datasets from TCGA (**A**) and GSE22138 (**B**).

Next, we performed a hierarchical clustering analysis of the pairwise correlations between the different immune subpopulations in UM samples from TCGA (Figure 4A). The heatmap revealed high correlations among several types of immune cells associated with cytotoxic T cell infiltration (i.e., lymphocytes, regulatory T cells, T follicular helper cells, M1 macrophages and CD8 T cells). We obtained similar results using Laurent microarray data (Figure 4B). Therefore, we concluded that the identified immune cells were significantly associated with immune infiltration in the tumor microenvironment.

We then examined the correlations between other immune parameters for UM patients in TCGA and Laurent UM dataset, and found a significant correlation between the immune score and the stromal score (Spearman’s r=0.806, 0.7789, respectively; Supplementary Figure 4A and 4B). We also observed a strong correlation between the immune score and T cell infiltration (Spearman’s r=0.7633, 0.4993, respectively; Supplementary Figure 4A and 4B). Additionally, a significant inverse correlation was found between the immune score and tumor purity (Spearman’s r =-0.9758, r=-0.9651 Supplementary Figure 4A and 4B). In the Laurent UM dataset, the association between the immune score and CD8 T cell infiltration remained significant (Supplementary Figure 4A and 4B), supporting the ability of the immune score to predict immune infiltration. Therefore, the immune score based on immune marker gene expression reflected immune cell infiltration in both the UM TCGA RNA-seq dataset and an independent microarray dataset.

### Prognostic value of immune cells and immune scores in TCGA and Laurent datasets

**Figure 5:**
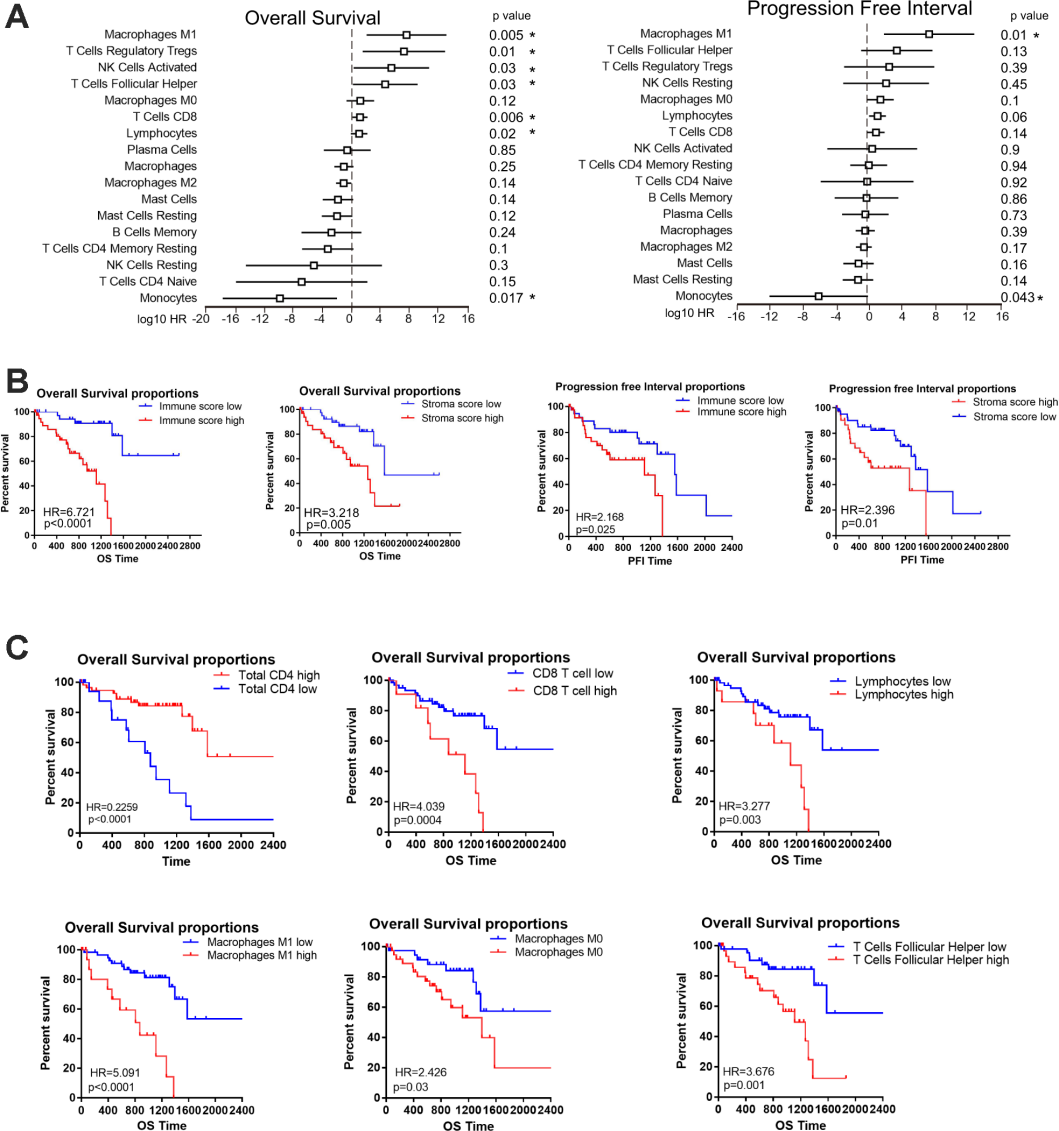
**The prognostic value of the immune score and immune cell infiltration in UM.** (**A**) HRs of OS and the PFI based on the infiltration of various immune cells (as continuous variables) in all patients (left); the horizontal bars represent the 95% confidence intervals of the HRs. Statistically significant variables are shown. Each cell type was evaluated individually and rank-ordered based on the estimated HR. (**B**) Kaplan-Meier survival analysis based on immune score and stroma score. Patients were divided into the high and low groups based on the level of immune score and stroma score. (**C**) Kaplan-Meier survival analysis based on selected immune cells. Patients were divided into the high and low groups based on their expression of each cell.

To assess the prognostic value of immune cells in UM, we first used a Univariate Cox regression model to investigate the associations of different immune subpopulations with the OS and PFI of patients in TCGA. The infiltration of M1 macrophages, activated natural killer cells and CD8 T cells was associated with a worse prognosis in the UM datasets (Figure 5A). On the contrary, the infiltration of monocytes was positively associated with patient survival (Figure 5A, 5B). In general, the infiltration of immune subpopulations involved in adaptive immunity is more likely to be an unfavorable prognostic factor than the infiltration of subpopulations involved in innate immunity.

To determine whether the immune score could predict the prognosis of UM patients in TCGA, we used a Kaplan-Meier curve and log-rank test to estimate the hazard ratios (HRs) of OS and the PFI. High immune scores and stromal scores were associated with worse OS in the datasets of TCGA (HR=6.721, P<0.0001, Figure 5B) and GSE22138 (HR=2.508, P=0.02, Supplementary Figure 1A). Next, we determined the associations of different immune cells with the patient prognosis. Consistent with the Univariate Cox regression analysis, our survival analysis revealed that particular immune subpopulations (M1 and M0 macrophages, CD8 T cells and T follicular helper cells) were associated with a worse prognosis, whereas total CD4 cells were associated with a better prognosis in the UM dataset of TCGA. Similar results were obtained with the GSE22138 dataset (Supplementary Figure 1B). These results suggest that UM patients with greater infiltration of immune cells and effector molecules exhibit poorer survival and may benefit from immunotherapies.

### Immune marker genes predict the prognosis of UM patients

**Figure 6:**
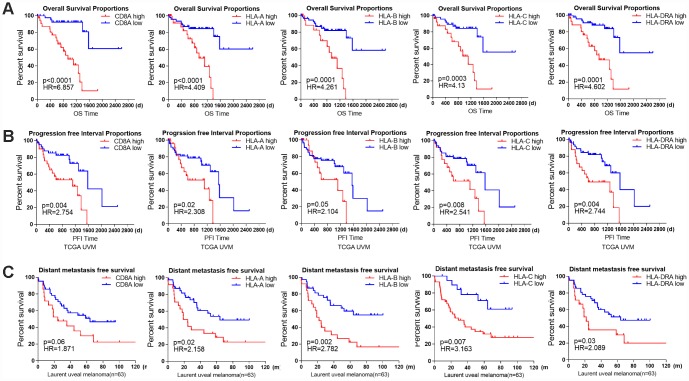
**The prognostic value of individual immune genes in UM.** (**A**) Kaplan-Meier survival curves demonstrate that elevated levels of CD8A (P<0.0001), HLA-A (P<0.0001), HLA-B (P=0.0001), HLA-C (P=0.0003) and HLA-DRA (P=0.0001) were consistently associated with worse OS in the UM dataset of TCGA. (**B**) Kaplan-Meier survival curves demonstrate that elevated levels of CD8A, HLA-A, HLA-B, HLA-C and HLA-DRA were consistently associated with a worse PFI in the UM dataset of TCGA (log-rank test, P<0.05). (**C**) Kaplan-Meier survival curves demonstrate that elevated levels of CD8A (P=0.06), HLA-A (P=0.02), HLA-B (P=0.002), HLA-C (P=0.007) and HLA-DRA (P=0.03) were consistently associated with worse metastasis-free survival in the Laurent UM dataset (n=63; log-rank test, P<0.05).

Next, we examined the prognostic value of individual immune genes in predicting patient survival. Higher mRNA levels of *CD8A* (P<0.0001), *HLA-A* (P<0.0001), *HLA-B* (P=0.0001), *HLA-C* (P=0.0003) and *HLA-DRA* (P=0.0001) in TCGA samples were associated with significantly shorter OS and PFIs (Figure 6A, 6B). Similarly, higher levels of *HLA-A* (P=0.02), *HLA-B* (P=0.002), *HLA-C* (P=0.007) and *HLA-DRA* (P=0.03) were associated with significantly shorter metastasis-free survival in the 63 patients in the Laurent UM dataset (Figure 6C), and *CD8A* expression (P=0.06) displayed a similar but non-significant trend of association with metastasis-free survival.

## DISCUSSION

TCGA has illuminated the genomic data from bulk tumor samples, and has provided detailed information about the tumor immune microenvironment [1, 24–26]. In previous studies, low immune cell infiltration has been associated with poor clinical outcomes for patients with different cancers [1, 27]. Through gene expression profiling, researchers can identify prognostic gene signatures and detect candidate genes for targeted therapies [27]. For example, an immune score based on gene expression data was found to correlate significantly with recurrence-free survival in thyroid cancer patients, regardless of their BRAF(V600E) status [28]. The fraction of immune cells in clinical tumor samples can be evaluated by multiple algorithms; indeed, aside from curating samples and performing basic pathologic characterization, investigators can analyze digitized hematoxylin & eosin-stained images of TCGA samples for tumor-infiltrating lymphocytes [3]. Using TCGA data, we identified three distinct immune subtypes of UM, with prognostic implications for immunological cancer management. The immune score was strongly associated with immune infiltration and poor outcomes, regardless of the tumor genome ploidy of the UM tumor samples. The underlying mechanism needs to be explored.

Genomic and transcriptomic data have been used to detect immune infiltration and to determine the molecular subtypes of ovarian cancer, melanoma and pancreatic cancer [29–31]. For instance, DNA sequencing data have been used to connect the neoantigen load to the T cell response and to link somatic mutations to immune infiltration [1, 32]. More recently, deconvoluted expression data have been used to measure the cytolytic activity in the tumor microenvironment and to quantify the infiltration of individual immune cell subsets [33–35]. A common theme across these studies is the integration of several types of genomic and clinical data, allowing for associations to be made among immune activity, gene expression, the mutation burden and patient survival. In this study, we examined the immune infiltration of UM samples through a single-sample GSEA and deconvolution method based on publicly available data in TCGA and the Gene Expression Omnibus. Our results suggested that the infiltration of immune cells differs markedly among immune subtypes. This analysis may ultimately reveal prognostic gene signatures and provide candidate genes for targeted therapies.

Despite the great success of immunotherapies against metastatic and late-stage melanoma [36, 37], immunotherapy has had limited success in UM [38]. UM is considered to be an immunotherapy-resistant subtype of melanoma, and UM patients are frequently excluded from clinical trials of immunotherapies for metastatic melanoma [20, 39, 40]. Although higher cytotoxic expression patterns are associated with better anti-tumor response and better patient survival in many solid tumors [34, 41, 42], the prognostic effects of immune infiltration depend on the type of tumor, the location of the cells and the state of activation. In UM, high levels of immune cells are associated with poor prognostic factors, such as M3 and *BAP1* mutation [15, 17, 43]. Immune cell infiltration occurs more frequently in epithelioid-cell-type UM, which also has a poor prognosis [44]. Crosstalk in the tumor microenvironment can promote the inflammatory response in cancer cells. Cancer cells may also promote the type 2 differentiation of macrophages and neutrophils, and may attract myeloid-derived suppressor cells and regulatory T cells to tumor sites. Thus, we speculate that UM cells may utilize immune cells for their survival and protection from immunological attack. The immunomodulatory microenvironment in the liver could further protect escaped UM cells from systemic immune surveillance [5, 44, 45].

De Lange et al have used unsupervised clustering to investigate the gene expression profiles of 64 enucleated eyes from UM patients, and divided them into class I tumors with a good prognosis and class IIa and IIb tumors with a poor prognosis [46]. Their study revealed an immune phenotype with a different prognosis. High expression of immune-related genes in class IIb UM suggested that the tumors were inflamed. Furthermore, study from TCGA of UM showed that the genes encoding chemotactic signals (e.g., CXCL9 and CXCL13), MHC class I (A, B, C) and MHC class II (DP, DM, DOA, DOB, DQ and DR) were upregulated in M3 patients [22]. Consistent with the previous studies, we demonstrated that *BAP1* inactivation was associated with immune infiltration and immune marker gene set expression, indicating the *BAP1* may regulate tumor immunology. GSVA results suggested that IRF4 targets and BCR pathways may be induced in *BAP1*-deficient tumors. Loss of *BAP1* expression is also associated with an increased infiltration of T cell follicular helper, Treg and CD8+ T cells, suggesting an inflammatory tumor microenvironment. Our data demonstrated that the immune cell subpopulations were differentially distributed between M3/BAP1null and D3/BAP1intact tumors, suggesting that BAP1 null tumors might be prioritized for immune checkpoint blockade therapies in UM.

At the time of this work, large, publicly available gene expression profiling datasets of UM patients treated with immune checkpoint blockers were not available. In addition, clinical trials in immunotherapy are being deployed earlier in the course of the disease, whereas the cohort in TCGA is more representative of the clinical population. We hope that large sequencing data from UM patients undergoing immune checkpoint blocker treatment will emerge in the future. Nevertheless, our analysis of the available datasets has advanced the application of genomic data to tumor immunology. The immune features reported herein should be considered for integration into prognostic models, or explored as predictors of adjuvant immune therapy responsiveness in patients with *BAP1*-deficient UM.

## MATERIALS AND METHODS

### UM gene expression datasets

RNA-seq data for UM samples were generated by TCGA and downloaded from the Genomic Data Commons Data Portal (https://portal.gdc.cancer.gov/). This dataset includes normalized gene expression profiles for 80 tumor samples in FPKM (Fragments Per Kilobase of transcript per Million fragments mapped). We downloaded one additional microarray gene expression dataset from the Gene Expression Omnibus database (accession number: GSE22138 [6]; n=63) to verify the immune scores and gene signatures and to predict the patients’ prognoses. GSE22138 included information on metastasis-free survival, while the UM dataset from TCGA included data on patients’ OS and PFI from the supplemental file of a previous study [1]. The clinical information and molecular data for the UM samples from TCGA were also downloaded from the supplemental file of a previous study [8]. The clinical information included the age, sex, metastatic status, histology cell type, American Joint Committee on Cancer clinical stage, mutation data, SCNA data and vital status of the patients.

We used the complete linkage method for hierarchical clustering analysis of the tumor samples, immune cell types and genes. The hierarchical clustering algorithm is agglomerative in that it joins samples based on a measure of multivariate distance, and prevents the joined samples from clustering independently again. Pair-wise joins in samples are represented as combined branches of a tree in a Dendrogram Plot. Pearson’s correlation distance method was used to determine whether samples clustered together [47].

### ESTIMATE

ESTIMATE is a tool that predicts tumor purity and detects infiltrating stromal/immune cells in tumor tissues based on gene expression data [23]. The ESTIMATE algorithm calculates the stromal and immune scores by performing single-sample GSEA for each sample. Data on the leukocyte fractions of the 80 tumor aliquots from TCGA were obtained from a previous study [1].

### CIBERSORT (immune cellular fraction estimates)

The relative fractions of 22 immune cell types within the leukocyte compartment were estimated with CIBERSORT (Cell-type Identification By Estimating Relative Subsets Of RNA Transcripts) [33]. CIBERSORT requires a specialized knowledgebase of gene expression signatures, termed a “signature matrix,” to deconvolute cell types of interest. CIBERSORT uses a set of 22 immune cell reference profiles (LM22) [33] to derive a base (signature) matrix that can be applied to mixed samples to determine the relative proportions of immune cells. LM22 is a signature matrix file consisting of 547 genes that can precisely distinguish 22 mature human hematopoietic populations (from peripheral blood or *in vitro* culture), including seven T cell types, naïve and memory B cells, plasma cells, natural killer cells, myeloid subsets, etc. LM22 can be applied to RNA-seq data as well as to microarray data. We used CIBERSORT to re-quantify several key immune gene signatures from GSE22138, and to identify the immune cells in the dataset from TCGA, as in a previous PanCancer immune study [1].

### Predicting patient survival with the Cox proportional hazards model

A Cox proportional hazards regression model was used to investigate the effectiveness of immune cells in predicting patients’ survival (PFI or OS). The optimal cut-off values of the proportions of immune cells in the cohort from TCGA were calculated based on their prognostic effects in X-Tile software [48]. The UM samples were then divided into high and low groups based on the cut-off point for the fraction level of each type of immune cell. In the subsequent scoring formula, the immune cell fraction level was given a value of 0 or 1. In STATA15, a univariate Cox proportional hazards model was used to evaluate the effects of significant prognostic immune cell fractions on survival outcomes. Kaplan-Meier plots were used to visualize the differences in survival between groups. The survival proportions of the different groups were compared through a log-rank test.

### GSVA

GSVA was performed as previously described [49] with the curated gene set collection of the Molecular Signatures Database (Broad Institute, c2. CP CPG). We searched for significant pathway gene sets that were differentially enriched between the *BAP1*^null^ and *BAP1*^intact^ groups (FDR≤0.01). The top 10 immune-related pathways were selected and shown in the heatmap.

### GSEA

Gene set collections for canonical pathways (C2.CP canonical pathways) were downloaded from the Molecular Signatures Database version 6.2 (www.broad institute.org/gsea/msigdb/collections.jsp). Gene set enrichment scores were calculated [50] with GSEA package version 1.32.0 with RNA-seq parameters. Differential gene set enrichment was determined using the limma package. The thresholds for statistical significance are noted in the Results.

### Statistical methods

All statistical analyses in this study were performed with R version 3.2.0 (R Foundation for Statistical Computing, Vienna, Austria) and SPSS Statistics 22.0. Survival curves were generated by the Kaplan-Meier method, and the log-rank test was used to compare the survival curves. The thresholds for each Kaplan-Meier plot were determined based on the unique score distribution of each dataset in X-Tile software [48]. We used Spearman's rank correlation coefficients to evaluate the correlation between the immune score and immune cell infiltration. The immune scores and stromal scores stratified by subgroup were compared by one-way ANOVA. A two-tailed P-value <0.05 was considered statistically significant.

## Supplementary Material

Supplementary Figures
