# Supplementary material for: Immunological analyses reveal an immune subtype of uveal melanoma with a poor prognosis
Source: Aging (Albany NY). 2020 Jan 18;12(2):1446–64. doi: 10.18632/aging.102693 (PMC7053626; doi:10.18632/aging.102693)
Supplement: Supplementary Figures [file aging-12-102693-s001..pdf]

SUPPLEMENTARY FIGURES

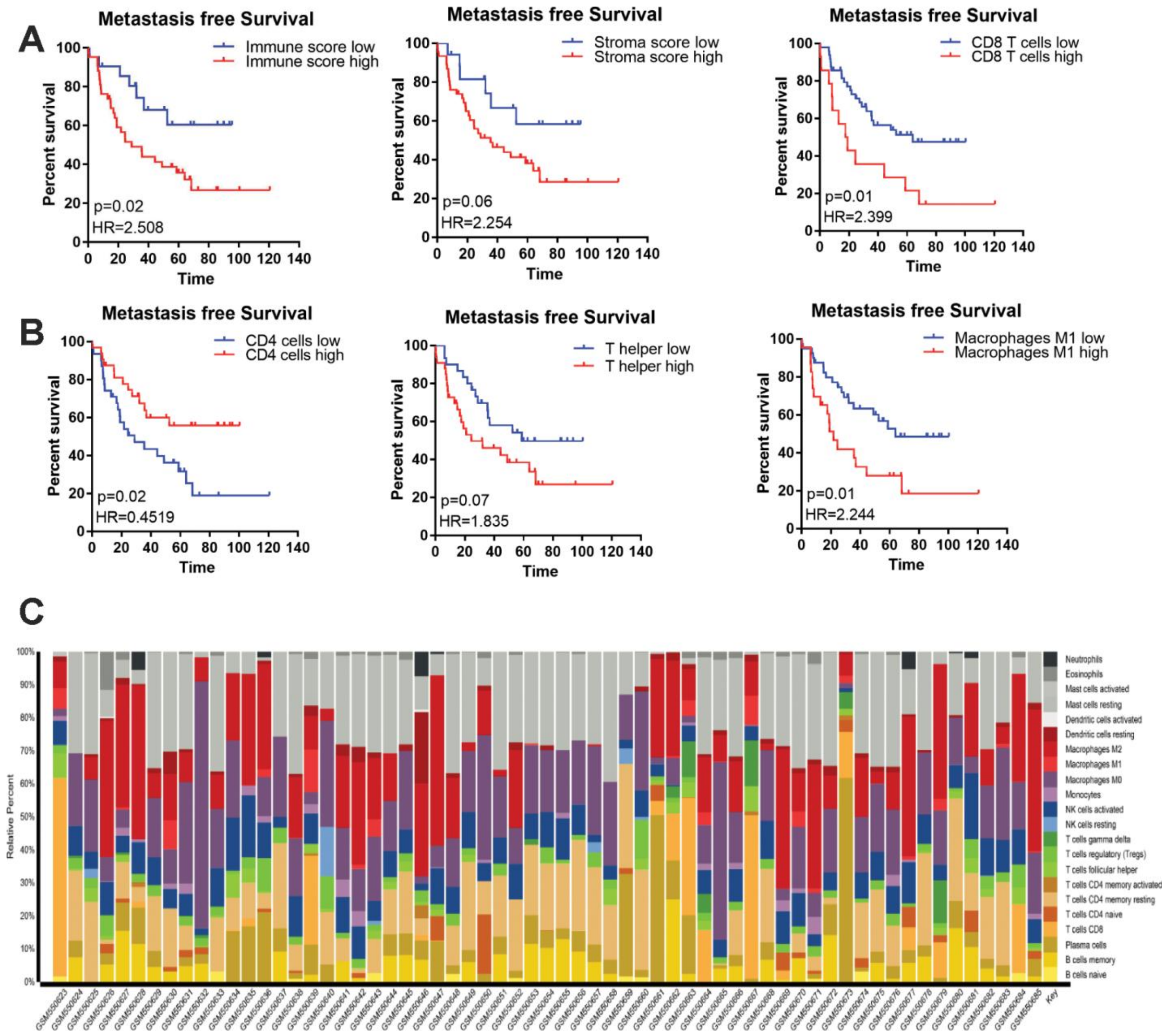

Supplementary Figure 1. The prognostic value of immune cell types in the Laurent UM dataset. (**A**) Kaplan-Meier survival analysis based on the immune score and stromal score. Patients were divided into high and low groups based on their value for each score. (**B**) Kaplan-Meier survival analysis based on immune cell types. Patients were divided into high and low groups based on their expression of each cell type. (**C**) The results of CIBERSORT in the Laurent UM dataset. n=63.

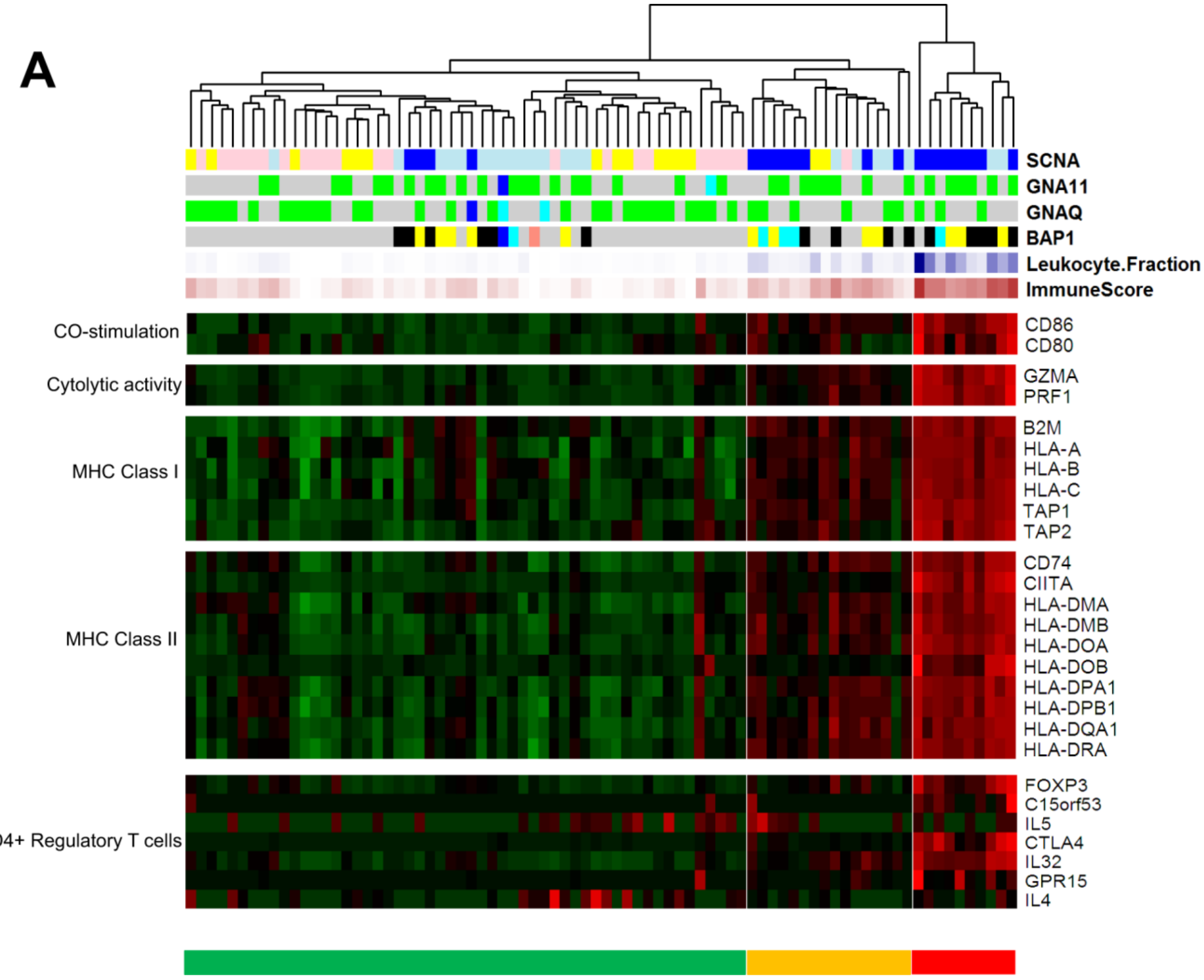

Supplementary Figure 2. Immune-related gene expression in the UM dataset of TCGA. (**A**) Hierarchical clustering of 80 tumors based on selected gene signatures or genes is shown.

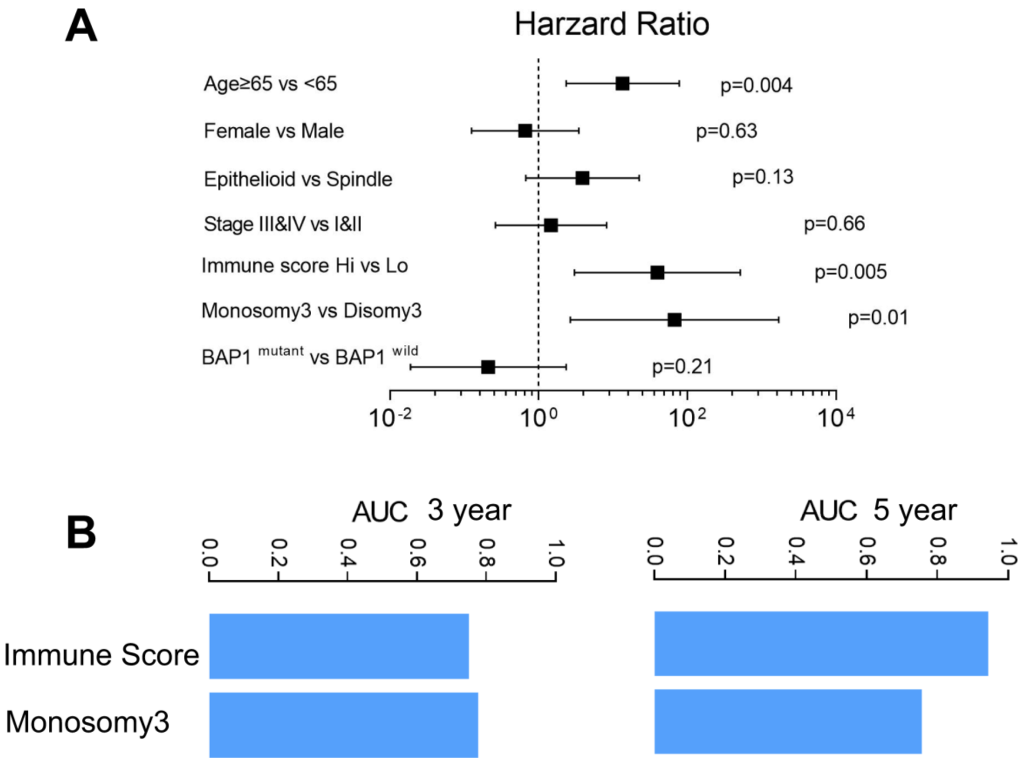

Supplementary Figure 3. The prognostic power of the immune score in the UM dataset of TCGA. (**A**) HRs based on various clinical features in all patients; the horizontal bars represent the 95% confidence intervals of the HRs. Statistically significant variables are shown. (**B**) Area under the curve for the immune score and M3 in predicting survival among UM patients in TCGA.

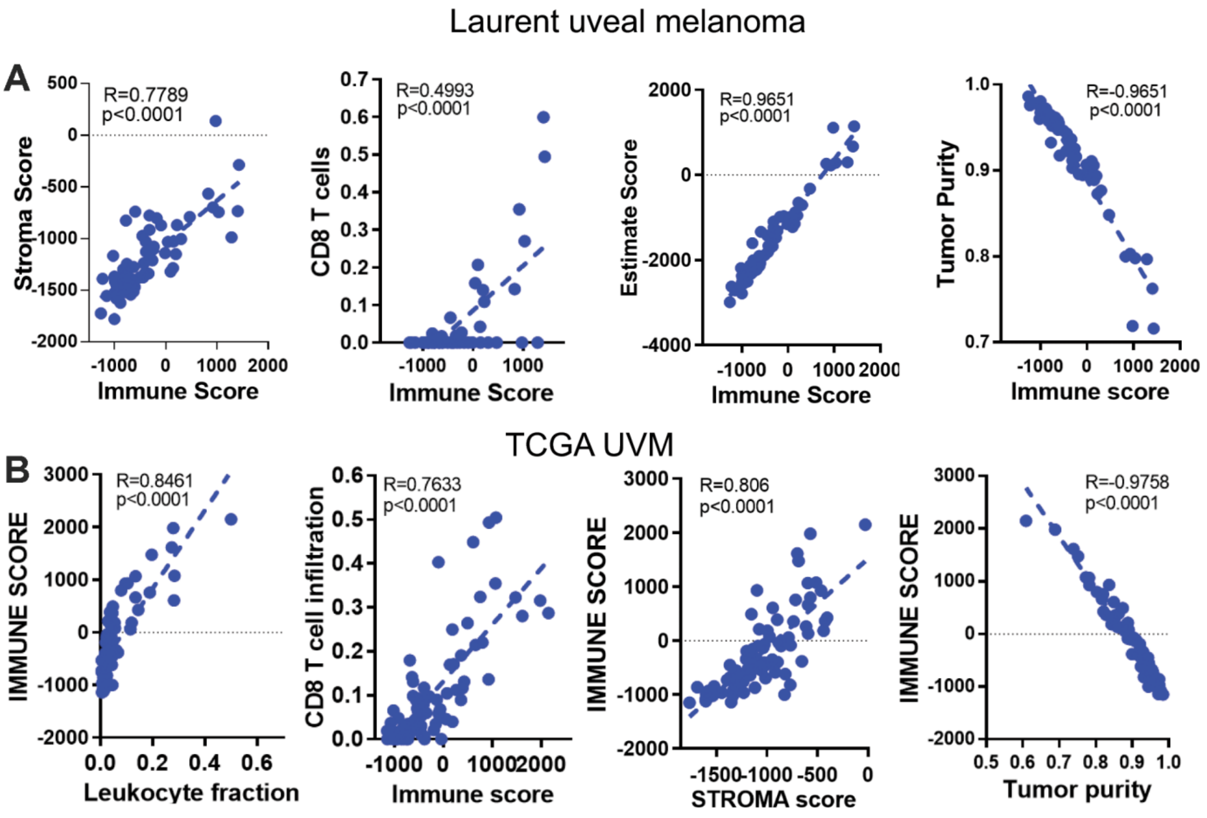

Supplementary Figure 4. The correlation between the immune score and immune cell infiltration in UM. (**A**) Correlations among the estimate score, CD8 T cell infiltration, stromal score, tumor purity and immune score in the GSE22138 dataset. (**B**) Correlations among CD8 T cell infiltration, the stromal score, tumor purity and the immune score in the UM dataset of TCGA.
